# Species Diversity of *Gymnopus* Section *Levipedes* in Southwestern China, with a Description of Three New Species

**DOI:** 10.3390/jof11020088

**Published:** 2025-01-23

**Authors:** Wei-Chao Feng, Xiao-Yan Li, Yang-Yang Cui, Qing Cai

**Affiliations:** 1 Key Laboratory of Phytochemistry and Natural Medicines, Kunming Institute of Botany, Chinese Academy of Sciences, Kunming 650201, China; fengweichao@mail.kib.ac.cn (W.-C.F.); lixiaoyan@mail.kib.ac.cn (X.-Y.L.); 2 Yunnan Key Laboratory for Fungal Diversity and Green Development, Kunming 650201, China; 3 College of Life Sciences, University of Chinese Academy of Sciences, Beijing 100049, China

**Keywords:** Agaricales, Omphalotaceae, phylogeny, morphology, three new taxa, taxonomy

## Abstract

Species of *Gymnopus* sect. *Levipedes* are challenging to delimitate due to the morphological similarity among different taxa. In this study, morphological characteristics, molecular phylogenetic data, and ecological traits were integrated to investigate the species diversity of this section of southwestern China. A total of 17 species were documented in the region, including three new species, namely *G*. *sinobrevipes*, *G. flavoalbus*, *G*. *yunnanensis*, and two species new to the studied area—*G. bicolor* and *G. ocior.* Detailed descriptions and illustrations of the three new species are presented, along with comparisons to closely related or morphologically similar species. The remaining species included five that were originally described from southwestern China, four that were first identified from Europe, two from the Republic of Korea, and one from North America. Six of these species, originally described outside of China, currently lack molecular evidence to support their distributions in southwestern China. Finally, 11 species with morphological and molecular evidence were recognized in southwestern China. A key to these species is also provided.

## 1. Introduction

*Gymnopus* (Pers.) Roussel belongs to Omphalotaceae Bresinsky with the type species *G*. *fusipes* (Bull.) Gray [1,2]. It is a widely distributed genus with over 200 species. The group was initially proposed as *Agaricus* trib. *Gymnopus* Pers [3], then elevated to genus *Gymnopus* (Pers.) Roussel in 1806 [4]. Several species of *Collybia* (Fr.) Staude were subsequently transferred to *Gymnopus*, and it was divided into three sections: *G*. sect. *Gymnopus* (Pers.) Roussel, *G*. sect. *Levipedes* (Quél.) Halling, and *G*. sect. *Vestipedes* (Fr.) Antonín, Halling and Noordel [5,6]. After that, the classification of the genus was revised several times [7,8,9]. According to the most recent comprehensive phylogenetic study, *Gymnopus* was divided into four sections: *G*. sect. *Androsacei* (Kühner) Antonín and Noordel., *G*. sect. *Gymnopus*, *G*. sect. *Levipedes*, and *G*. sect. *Impudicae* Antonín and Noordel [10].

*Gymnopus* sect. *Levipedes* is the most species-rich section of the genus. Species of this section are characterized by having a smooth, polished, or pubescent stipe, a pileipellis composed of inflated, lobed, or coralloid elements (Dryophila-type structure) [6,8,10]. It is challenging to define and recognize the species of this section due to the morphological similarities among different taxa [11,12,13]. As currently circumscribed, over 50 taxa have been described and accepted worldwide [6,12,14,15,16,17,18,19,20,21,22,23,24,25,26,27,28,29]. The majority of these taxa were first described from Europe, with a total of 20 species [3,12,15,16,19,20,21,24]. Additionally, 18 taxa were originally described from East Asia, and seven were described from North America [14,15,17,18,24,25,26,27,28,29]. In contrast, few investigations have been conducted in other areas of the world [15,17]. In recent years, 26 species of *G*. sect. *Levipedes* have been recorded in China. Of these, 16 species were originally described from China, seven from Europe, two from the Republic of Korea, and one from North America [25,26,27,28,29,30,31,32,33,34,35,36,37,38,39,40,41,42,43]. Most molecular phylogenetic and taxonomy studies related to this section in China have mainly concentrated on the northeastern region, where 16 species have been recorded [25,33,35,39,44]. In comparison, 12 species have been documented in southwestern China. Among these, four species were originally described from this region, four from Europe, two were first reported from the Republic of Korea, one originally described from central China, and one was originally described from North America. However, only six of these species have molecular evidence to confirm their distribution in southwestern China [26,28,29,31,34,36,37,40,41,42,43,45].

During our investigations of macrofungi in southwestern China, several specimens of *G*. sect. *Levipedes* were collected. In this study, we used morphological characteristics, molecular phylogenetic data, and ecological traits to elucidate the species diversity of *G*. sect. *Levipedes* in southwestern China and uncovered three undescribed species. They are described and illustrated below.

## 2. Materials and Methods

### 2.1. Taxon Sampling

Fifteen specimens of *G*. sect. *Levipedes* from southwestern China were examined. Among these, 11 specimens were collected in subtropical broad-leaved forests primarily dominated by trees by Ericaceae Juss, Fagaceae Dumort, Lauraceae Juss, and Theaceae Mirb. Three specimens were collected in coniferous forests consisting of *Picea* spp., *Abies* spp., and *Pinus armandii* Franch. The remaining specimen was collected from a mixed forest dominated by *Picea* spp. and *Quercus* spp. For each collection, a part of the basidioma was dried with silica gel for DNA extraction, and the remaining material was dried with an electronic food dehydrator at 45–50 °C. All specimens examined in this study were deposited in the Herbarium of the Kunming Institute of Botany, Chinese Academy of Sciences (KUN-HKAS). New taxa are registered with the Fungal Names repository, Institute of Microbiology, Chinese Academy of Sciences in Beijing, China.

### 2.2. Morphological Observation

The macroscopic descriptions are based on field notes and photographs of fresh basidiomata. Color codes in the descriptions were taken from RAL DESIGN SYSTEM plus color (second edition, 2023). Microscopic characteristics were studied with light microscopy using dried material rehydrated in 5% KOH and stained with Congo Red when necessary. Melzer’s reagent was used to check the amyloidity of basidiospores. In the description of basidiospores, the abbreviation (n/m/p) represents n basidiospores measured from m basidiomata of p collections. Dimensions for the basidiospores are given using a range notation of the form (a–) b–c (–d). The range b–c contains a minimum of 90% of the measured values. Extreme values, a or d, are given in parentheses. Q represents the ‘length/width ratio’ of a basidiospore in the side view, and Q_m_ represents the average Q of all basidiospores measured ± sample standard deviation.

### 2.3. DNA Extraction, PCR Amplification, and Sequencing

The genomic DNA was extracted from the silica gel-dried material or herbarium specimens using the Ezup Column Fungi Genomic DNA Purification Kit (Sangon Biotech, Shanghai, China). Two nuclear loci were sequenced, including the nuclear ribosomal internal transcribed spacer (ITS) and the large subunit of nuclear ribosomal RNA (nrLSU) using the primer pairs ITS1F/ITS4 [46] and LR0R/LR5 [47], respectively. PCR protocols were as follows: initial denaturation at 94 °C for 3 min, followed by 35 cycles of denaturation at 94 °C for 30 s, annealing at 51 °C to 53 °C 40 s for ITS and 48 °C 40 s for nrLSU, elongation at 72 °C 60 s for ITS and 90 s for nrLSU, and one final elongation at 72 °C for 8 min.

### 2.4. Sequence Alignments and Phylogenetic Analyses

The newly generated ITS sequences were blasted in GenBank, and the most closely related sequences (nucleotide identities > 95%) were retrieved to complement the ITS dataset with one to four representatives per species. The corresponding nrLSU sequences were also downloaded. According to the recent phylogenetic studies, five representative species of *G*. sect. *Impudicae* and *G*. sect. *Gymnopus* were selected as outgroups [10,29].

Sequences of the two gene fragments (ITS and nrLSU) were separately aligned with MAFFT *7* online [48,49] and manually adjusted with BioEdit 7.2.5.0 [50]. The aligned datasets were then concatenated using PhyloSuite v1.2.3 [51,52]. The best-fit substitution models were determined by MrModeltest 2.3 [53]. Maximum Likelihood (ML) and Bayesian Inference (BI) analyses were performed with raxmlGUI 2.0 [54] and MrBayes v.3.2.6 [55], respectively. In the ML analyses, a GTR-Gamma I model of evolution was employed, and bootstrap values were assessed through 1000 rapid bootstrap replicates. For BI analyses, four Markov Chain Monte Carlo (MCMC) chains were run simultaneously for 18 million generations under the best evolutionary models selected using MrModeltest, and trees were sampled every 1000 generations. Runs were automatically terminated when the average standard deviation of split frequency fell below 0.01. Chain convergence was also evaluated using Tracer v1.7.2 (http://tree.bio.ed.ac.uk/software/tracer/, accessed on 6 November 2024) to confirm all effective sampling size values were over 200. Subsequently, the sampled trees were summarized, and posterior probabilities were obtained by discarding the first 25% of generations as burn-in.

## 3. Results

### 3.1. Phylogenetic Analyses

The dataset contained 173 sequences, comprising 107 ITS sequences and 66 nrLSU sequences, and 30 of them were newly generated in this study. These sequences represent 107 samples of 59 taxa. The GenBank accession numbers of the sequences retrieved from GenBank and obtained in this study are listed in Table 1. The most suitable models selected by MrModeltest were GTR + G for the ITS sequences and HKY + I + G for the nrLSU sequences.

**Figure 1:**
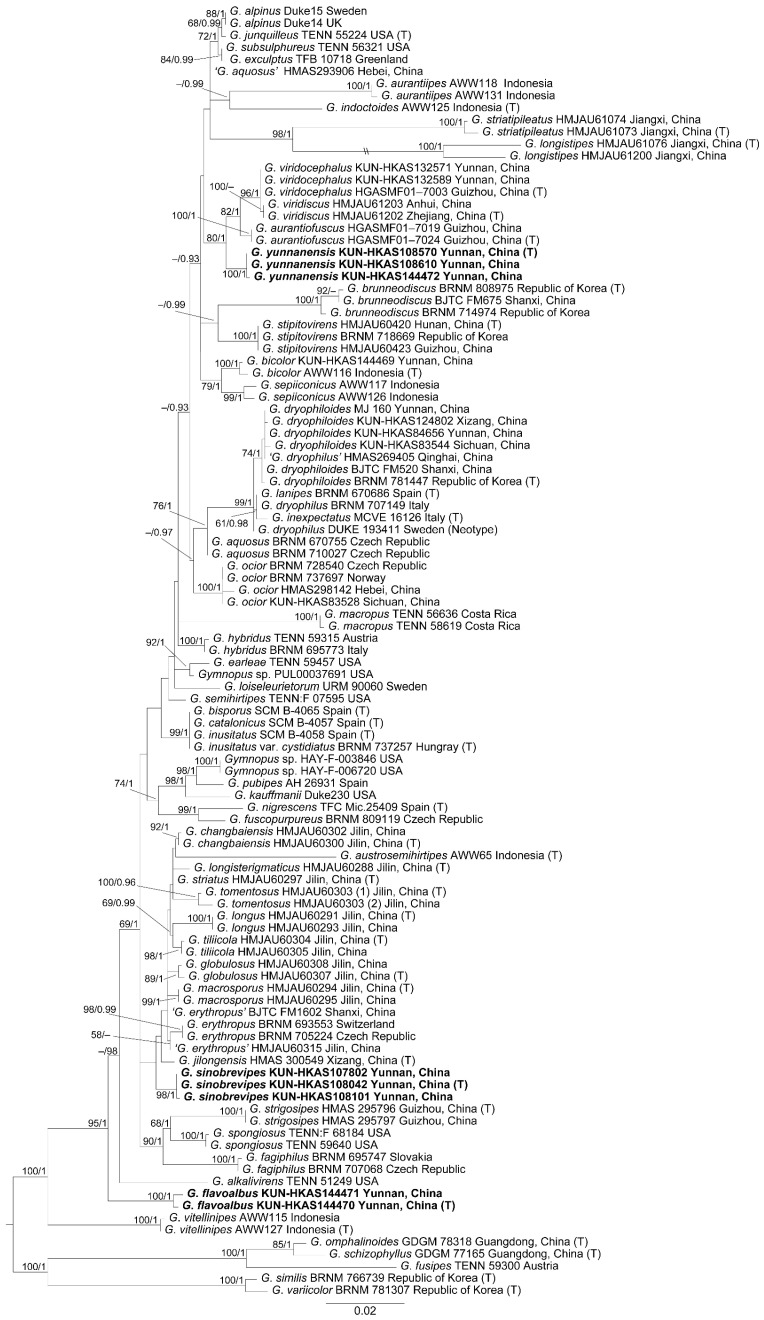
Phylogenetic tree of *Gymnopus* sect. *Levipedes* inferred from maximum likelihood analyses based on the ITS and nrLSU sequences. Bootstrap values ≥ 50 and Bayesian posterior probabilities ≥ 0.9 are shown along branches. Sequences from type collections are indicated with (T), and new species are highlighted in bold.

The phylogenetic trees inferred from the ML and BI analyses were similar in topology. Therefore, only the trees generated from the ML analyses were presented (Figure 1). The collections from southwestern China were clustered into 11 lineages in the phylogenetic tree. Eight of these lineages corresponded to known species, viz. *G*. *aurantiofuscus* J J.P. Li, Chang-Tian Li and Chun Y. Deng, *G. bicolor* A.W. Wilson, Desjardin and E. Horak, *G. dryophiloides* Antonín, Ryoo and Ka, *G. jilongensis* Ke Wang, T.Z. Wei and P. Hong, *G. ocior* Antonín and Noordel., *G*. *stipitovirens* J.P. Li, Chang-Tian Li and Antonín, *G*. *strigosipes* J.P. Li, Chang Tian Li, Yi Li and Yu Li, and *G. viridocephalus* J.P. Li, Chang-Tian Li and Chun Y. Deng. Based on our molecular phylogenetic analyses and morphological studies, the remaining three lineages were described as new species, namely *G*. *sinobrevipes*, *G. flavoalbus*, and *G*. *yunnanensis*.

### 3.2. Taxonomy

**Figure 2:**
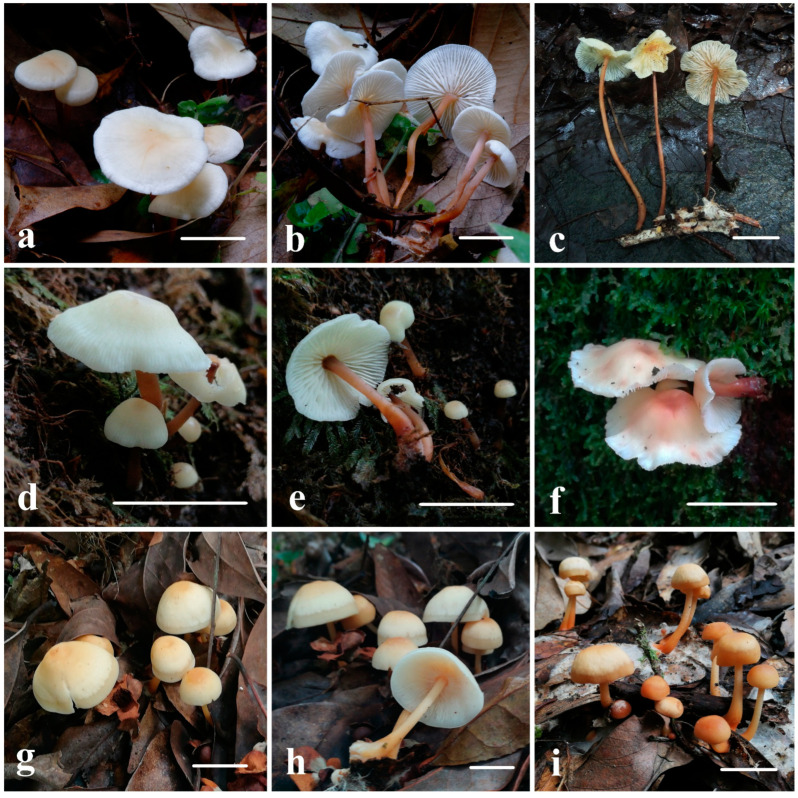
Basidiomata of novel species in *Gymnopus* sect. *Levipedes* from southwestern China. (**a**–**c**) *G*. *flavoalbus* ((**a**,**b**) Holotype, KUN-HKAS144470, (**c**) KUN-HKAS144471); (**d**–**f**) *G*. *sinobrevipes* ((**d**,**e**) Holotype, KUN-HKAS108042, (**f**) KUN-HKAS108101); (**g**–**i**) *G*. *yunnanensis* ((**g**,**h**): Holotype, KUN-HKAS108570, (**i**) KUN-HKAS108610). Bars = 2 cm.

**Figure 3:**
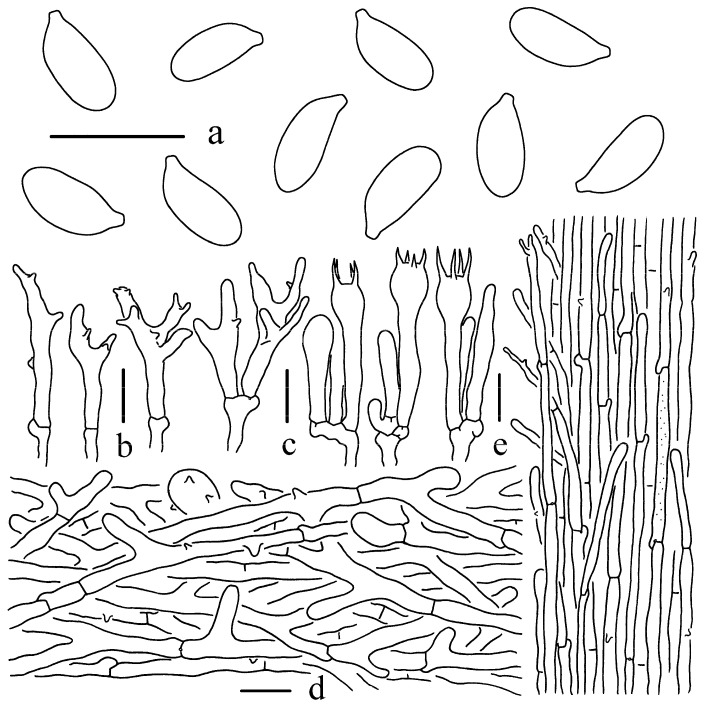
Microscopic features of *G*. *flavoalbus* (Holotype, KUN-HKAS144470). (**a**) Basidiospores; (**b**) Cheilocystidia; (**c**) Basidia; (**d**) Pileipellis; (**e**) Stipitipellis. Bars: (**a**–**c**) = 10 μm, (**d**,**e**) = 20 μm.

*Gymnopus flavoalbus* W.C. Feng, Y.Y. Cui and Q. Cai, sp. nov., Figure 1, Figure 2 and Figure 3.

Fungal Name: FN 571794.

Etymology: Referring to the pale yellow center and whitish margin of the pileus.

Diagnosis: Similar to *G. macropus* Halling, but differs by its light-colored pileus, furcate cheilocystidia, and pileipellis with broadly clavate to spherical terminal cells without pigments.

Type: China. Yunnan Province: Baoshan City, Longyang District, 25°18′41″ N, 98°46′13″ E, altitude 1980–2415 m, 2 June 2022, GLG-FXP40 (Holotype, KUN-HKAS144470, GenBank Acc. Nos.: ITS =PQ771152, nrLSU = PQ771137).

Description: Pileus 12–36 mm, hemispherical to plano-convex when young, margin clearly involute, becoming applanate with age, sometimes remaining slightly involute, broadly convex at center, pale yellow (RAL 070 90 10, 070 85 20), darker when wet, slightly lighter towards margin, off-white (RAL 090 90 10, 090 90 05), margin pure white (RAL 9010), striations radiating from the disc when mature. Context white (RAL 9010), thin, spongy. Lamellae adnate to adnexed, ventricose, edge transparent when dry, dull white (RAL 9010), occasionally apricot pink (RAL 030 85 10, 010 90 10) in certain spots. Stipe 40–93 × 2–5 mm, slender, hollow, cylindrical, often twisted, upper part pinkish brown (RAL 010 70 15) to pale purple (RAL 010 70 10), lower part rusty brown (RAL 040 50 40, 040 50 30), or dull brown (RAL 050 60 40, 050 50 30) to rusty brown (RAL 040 50 30), smooth or sparsely white tomentum (RAL 9010), base with white (RAL 9010) mycelium. Odor and taste indistinct.

Basidiospores [46/2/2] 6–8 × 3–4 μm, Q= (1.79–)1.83–2.21(–2.35), Qm = 2.03 ± 0.13, oblong, subcylindrical, smooth, hyaline, thin-walled, inamyloid. Basidia 22–34 × 4–8 μm, four-spored, narrowly clavate to clavate, often with one to two constrictions, thin-walled, hyaline. Lamellar trama composed of subregular, hyaline, thin-walled hyphae 2–8 μm wide. Lamellar edge sterile. Pleurocystidia not observed. Cheilocystidia numerous, 16–33 × 3–8 μm, narrowly cylindrical, narrowly clavate, 2–3 obtuse branched at the apex, furcate and lobate, with more diverticula, rarely obtuse with filiform apical projections. Pileipellis a cutis composed of strongly intertwined hyphae, 3–11 μm wide, cylindrical or with short branches and diverticula; terminal cells irregular cylindrical or shallowly lobed at apex, 17–40 × 4–8 μm, sometimes broadly clavate to spherical, up to 27 μm wide, with diverticula, thin-walled, hyaline. Stipitipellis consisting of longitudinally arranged, thin-walled hyphae, 2–8 μm wide, few hyphae with yellowish green (RAL 090 85 30) pigments; terminal cells narrowly cylindrical, apex obtuse, 20–90 × 2–4 μm, with filamentous appendages or small diverticula, thin-walled, hyaline. Clamp connections present in all tissues.

Habitat: Scattered on fallen leaves or dead branches in subtropical broad-leaved forest.

Distribution: Known from southwestern China.

Additional specimens examined: China. Yunnan Province: Baoshan City, Tengchong County-level City, 25°16′35″ N, 94°40′22″ E, altitude 1730–2170 m, 6 June 2022, GLG-LFF65 (KUN-HKAS144471).

Notes: *Gymnopus flavoalbus* is characterized by its small basidioma with a pale yellow pileus that is often pure white at the margin, abundant cheilocystidia, and the terminal cells of the pileipellis that are sometimes broadly clavate to spherical. Based on our phylogenetic analysis, *G*. *flavoalbus* forms a distinct lineage without any closely related species (Figure 1). Morphologically, *G*. *flavoalbus* looks like *G. macropus* and *G. indoctoides* A.W. Wilson, Desjardin, and E. Horak due to their light brown to brown pileus with a whitish margin and slender stipes [15,17]. However, *G*. *macropus*, originally described from Colombia, has dark-colored pileus, rarely bifurcate cheilocystidia, pileipellis hyphae that contain alkali-insoluble, encrusting brown pigments when young, and lacks broadly clavate to spherical terminal cells [15]. *Gymnopus indoctoides*, originally described from Indonesia, differs from *G. flavoalbus* by its smaller basidiospores (4.8–6.4 × 2–3.2 μm), and the absence of broadly clavate to spherical terminal cells in the pileipellis [17]. In addition, *G*. *globulosus* J.J. Hu, Y.L. Tuo, B. Zhang and Yu Li, described from northeastern China, can be also confused with *G*. *flavoalbus* because their terminal hyphae in the pileipellis are inflated to spherical to prolate [26]. However, *G*. *globulosus* can be distinguished from *G*. *flavoalbus* by its larger basidioma and basidiospores (measuring 7.0–8.8 × 3.3–4.2 μm) [26].

**Figure 4:**
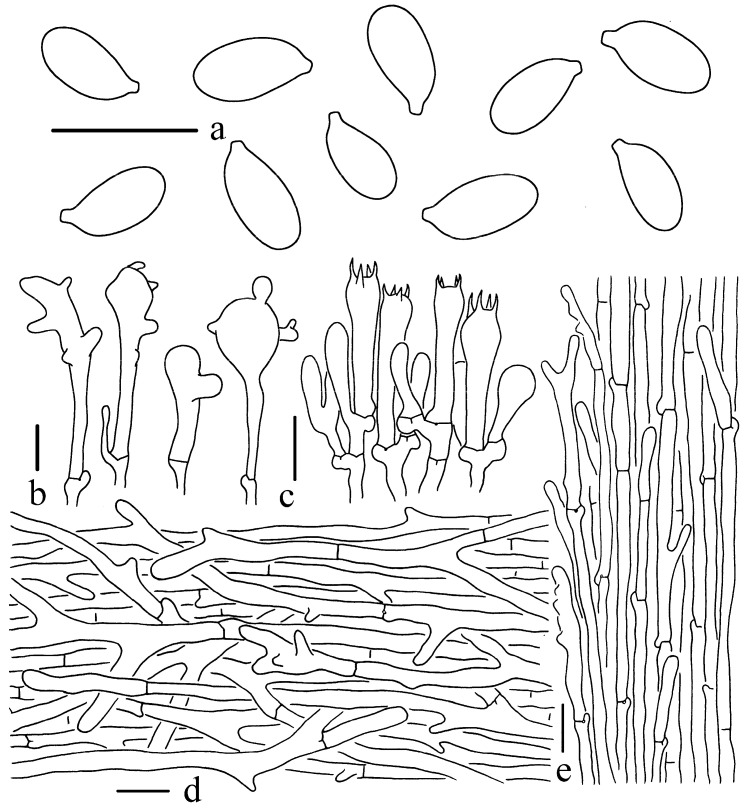
Microscopic features of *G*. *sinobrevipes* (Holotype, KUN-HKAS108042). (**a**) Basidiospores; (**b**) Cheilocystidia; (**c**) Basidia; (**d**) Pileipellis; (**e**) Stipitipellis. Bars: (**a**–**c**) = 10 μm, (**d**,**e**) = 20 μm.

*Gymnopus sinobrevipes* W.C. Feng, Y.Y. Cui, and Q. Cai, sp. nov., Figure 1, Figure 2 and Figure 4.

Fungal Name: FN 571796

Etymology: Referring to the species from China and having short stipe.

Diagnosis: Similar to *G. tiliicola* J.J. Hu, B. Zhang, and Yu Li, but differs in its smaller pileus and cheilocystidia with knobs.

Type: China. Yunnan Province: Honghe Hani and Yi Autonomous Prefecture, Hekou Yao Autonomous County, 25°8′10″ N, 102°44′29″ E, altitude 2075 m, 14 September 2019, Caiqing-532532MF0420 (Holotype, KUN-HKAS108042, GenBank Acc. Nos.: ITS =PQ771149, nrLSU = PQ771134).

Description: Pileus hemispherical to oblate when young, then campaniform to hemispherical with reflexed margin at maturity, 10–30 mm in diam., obviously depressed at the center, pale yellow (RAL 100 70 20, 095 85 20) or yellowish brown (RAL 085 80 20, 080 80 20, 080 70 20), turning reddish brown (RAL 040 85 20, 040 80 20, 030 60 40, 030 60 30) with age, margin undulate and with distinctive striations, pure white (RAL 9010) or off-white (RAL 090 93 05). Context white (RAL 9010) to creamy white (RAL 090 93 05), thin. Lamellae adnate to adnexed, ventricose, concolorous as pileus margin, occasionally pinkish white (RAL 010 90 10, 010 60 20) in the region near stipe when young, with reddish brown (RAL 030 60 40, 030 60 30) tones in some areas at maturity. Stipe 15–40 × 1–3 mm, cylindrical, apex beige (RAL 070 90 10, 070 85 20) to pale yellowish brown (RAL 070 90 20, 070 80 20) and lower part darker brown (RAL 070 60 30, 060 60 30, 060 50 40, 060 50 30) when young, when mature becoming pale reddish brown (RAL 030 60 30), reddish brown (RAL 030 50 30, 030 50 20) to brick brown (RAL 040 40 30, 030 40 30), hollow, base with white (RAL 9010) tomentum. Odor and taste indistinct.

Basidiospores [60/2/3] 6–7.5 (–8.5) × (3–) 3.5–4 μm, Q = (1.76–) 1.8–2.07 (–2.12), Qm = 1.93 ± 0.09, ellipsoid to subcylindrical, smooth, thin-walled, hyaline, inamyloid. Basidia 20–26 × 3–6 μm, narrowly cylindrical to narrowly clavate, mostly four-spored, occasionally two-spored, thin-walled, hyaline. Lamellar trama subregular, hyphae 2–10 μm wide, thin-walled, hyaline. Lamellar edge sterile. Cheilocystidia 22–43 × 6–13 μm, irregular clavate, narrowly clavate to clavate, subcapitate to captitate, with knobs, wide projections, hyaline, thin-walled. Pleurocystidia not observed. Pileipellis a cutis, composed of parallel and slightly interwoven hyphae, 2–9 μm wide, sometimes with short branches or projections; terminal cells (15–) 30–70 × 3–8 μm, irregular cylindrical, mostly furcate, or with diverticula, thin-walled, hyaline. Stipitipellis composed of thin-walled, longitudinally arranged cylindrical hyphae 2–10 μm wide, occasionally diverticulate; terminal cells 23–63 (100) × 3–7 μm, narrowly cylindrical to narrowly clavate, apex obtuse or short-furcate at the apex, irregular clavate with knobs, arranged in wavy pattern, thin-walled, hyaline. Clamp connections present in all tissues.

Habitat: Scattered at the base of trees or on the leaf litter in subtropical broad-leaved forest.

Distribution: Known from southwestern China.

Additional specimens examined: China. Yunnan Province: Honghe Hani and Yi Autonomous Prefecture, Hekou Yao Autonomous County, 25°8′09″ N, 102°44′29″ E, altitude 2040 m, 21 August 2022, Caiqing-532532MF0156 (KUN-HKAS107802); same location, 25°8′09″ N, 102°44′29″ E, altitude 2040 m, 14 September 2019, Xuxin-532533MF0483 (KUN-HKAS108101).

Notes: *Gymnopus sinobrevipes* is characterized by its smaller basidiomata, campaniform to hemispherical pileus when mature, and the presence of variable cheilocystidia. In the phylogenetic analyses, *G. sinobrevipes* is related to the clade formed by *G. austrosemihirtipes* A.W. Wilson, Desjardin, and E. Horak, *G*. *changbaiensis* J.J. Hu, B. Zhang, and Yu Li, *G*. *erythropus* (Pers.) Antonín, Halling, and Noordel., *G*. *globulosus* J.J. Hu, Y.L. Tuo, B. Zhang, and Yu Li, *G*. *jilongensis*, *G*. *longisterigmatus* J.J. Hu, B. Zhang, and Yu Li, *G*. *longus* J.J. Hu, B. Zhang, and Yu Li, *G*. *macrosporus* J.J. Hu, B. Zhang, and Yu Li, *G*. *tomentosus* J.J. Hu, B. Zhang, and Yu Li, *G*. *tiliicola*, and *G*. *striatus* J.J. Hu, B. Zhang, and Yu Li (Figure 1). Most species in the clade were described from China, with the exception of *G. austrosemihirtipes* which was described from Indonesia and *G*. *erythropus* described form Europe. *Gymnopus austrosemihirtipes* can be clearly distinguished from *G*. *sinobrevipes* by its brown stipe, the presence of basidiole-like cheilocystidia and weakly annular-incrusted hyphae in the pileipellis [17]. *Gymnopus longisterigmatus*, *G*. *longus,* and *G*. *macrosporus*, collected in temperate mixed forests with coniferous and broad-leaved trees, can be easily distinguished from *G. sinobrevipes* by their basidia with extremely longer sterigmata (up to 32–40 μm) [25]. *Gymnopus sinobrevipes* differs from *G*. *erythropus*, *G*. *jilongensis,* and *G*. *striatus* by its basidiomata with shorter and thinner stipes; otherwise, *G. sinobrevipes* was found in subtropical broad-leaved forests, while the latter three taxa were found in temperate coniferous forests or mixed forests of coniferous and broad-leaved trees [3,25,28]. *Gymnopus tomentosus* can be distinguished from *G. sinobrevipes* by its pileus with a tomentum margin and the presence of inflated bulbous terminal hyphae in the pileipellis [25]. *Gymnopus globulosus* differs from *G*. *sinobrevipes* by its larger basidiospores and two-layered pileipellis, with the upper layer composed of spherical to prolate hyphae and the lower layer possessing branched and inflated hyphae with light brown to brown incrustation [25]. *Gymnopus sinobrevipes* looks like *G*. *tiliicola* and *G. changbaiensis,* but *G*. *tiliicola* has a larger pileus (measuring 30–67 mm), clavate cheilocystidia without knobs and wide projections, and was collected at the base of *Tilia* sp. in northeastern China; *Gymnopus changbaiensis* has a longer stipe (measuring 42–53 mm), clavate cheilocystidia without knobs and wide projections, and was found in the temperate mixed forests with coniferous and broad-leaved trees in northeastern China [25].

**Figure 5:**
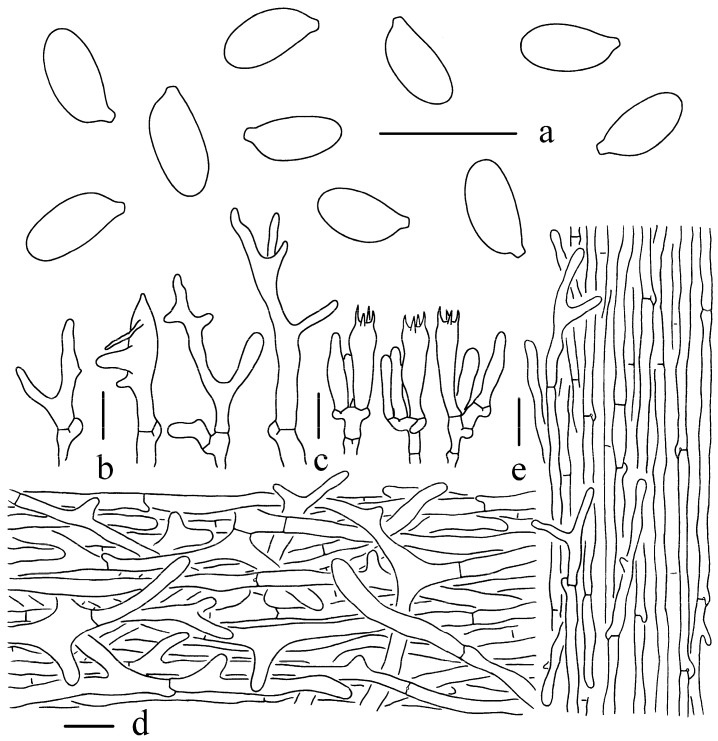
Microscopic features of *G*. *yunnanensis* (Holotype, HKAS108570). (**a**) Basidiospores; (**b**) Cheilocystidia; (**c**) Basidia; (**d**) Pileipellis; (**e**) Stipitipellis. Bars: (**a**–**c**) = 10 μm, (**d**,**e**) = 20 μm.

*Gymnopus yunnanensis* W.C. Feng, Y.Y. Cui and Q. Cai, sp. nov., Figure 1, Figure 2 and Figure 5.

Fungal Name: FN 571797

Etymology: Referring to the locality, Yunnan Province, China, where the holotype was collected.

Diagnosis: Similar to *G. dryophiloides*, but differs in its oblong to subcylindrical basidiospores and the absence of rhizomorphs at the stipe base.

Type: China. Yunnan Province: Honghe Hani and Yi Autonomous Prefecture, Lvchun County, 22°59′20″ N, 102°26′24″ E, altitude 1660 m, 23 September 2019, Xuxin-532531MF0478 (Holotype, KUN-HKAS108570, GenBank Acc. Nos.: ITS =PQ771154, nrLSU = PQ771139).

Description: Pileus 15–65 mm in diam., oblate or campaniform when young, with a slight depression in the center, then becoming applanate, with an umbo at center, clearly hygrophanous and translucent at 1/4 of the margin, sometimes wavy and margin slightly inflexed, glabrous, pale yellow-brown (RAL 085 80 20, 080 85 30) to brownish yellow (RAL 075 80 30, 070 70 30) at center, turning milky white (RAL 110 96 02, 110 93 05, 090 93 05) from the disc towards margin, strongly pallescent on drying, yellowish white (RAL 090 90 20, 080 90 10), darker in disc or dull yellowish brown (RAL 070 60 50, 070 60 40, 070 60 30), margin still clearly hygrophanous. Context creamy white (RAL 090 93 05), thin. Lamellae adnate, ventricose, closely spaced, white (RAL 9010) to milky white (RAL 090 93 05), sometimes undulate. Stipe 30–90 × 2–5 mm, cylindrical or slightly broader at base, hollow, yellowish white (RAL 090 80 30, 090 70 30, 085 80 30), darker below, yellowish brown (RAL 085 70 30, 080 60 40), sparsely covered with minute white tomentum (RAL 9010), base with white (RAL 9010) and wiry mycelium. Odor and taste indistinct.

Basidiospores [60/3/3] (4.5–) 5–6.5 (–7) × 2.5–3 (–3.5) μm, Q = (1.68–) 1.72–2.10 (–2.13), Qm = 1.91 ± 0.11, oblong, occasionally subcylindrical, thin-walled, smooth, hyaline, inamyloid. Basidia 16–22 × 4–7 μm, four-spored, clavate to narrowly clavate, thin-walled, hyaline. Lamellar edge sterile. Cheilocystidia abundant and variable, 15–57 × 2–6 μm, irregular, furcate, with blunt and tiny projections, occasionally narrowly cylindrical or subfusoid, sometimes with filiform excrescences, thin-walled, colorless. Pleurocystidia absent. Lamellar trama subregular, composed of thin-walled, cylindrical hyphae 2–11 μm wide. Pileipellis a cutis, composed of thin-walled, slightly interwoven hyphae 3–12 μm wide, sometimes with short branches and projections; terminal cells narrowly cylindrical, apex often bifid, 24–68 × 3–12 μm, hyaline. Stipitipellis composed of thin-walled, longitudinally arranged hyphae, 2–6 μm wide, occasionally with diverticula; terminal cells irregular cylindrical to clavate, curved, apex often furcate, or with short branches, 30–65 × 3–5 μm, thin-walled, hyaline. Clamp connections present in all tissues.

Habitat: Scattered or gregarious on leaf litter in subtropical broad-leaved forest.

Distribution: Known from southwestern China.

Additional specimens examined: China. Yunnan Province: Honghe Hani and Yi Autonomous Prefecture, Lvchun County, 22°59′56″ N, 102°26′31″ E, altitude 2190 m, 23 September 2019, Caiqing-532531MF0531 (KUN-HKAS108610); Nujiang Lisu Autonomous Prefecture, Lushui City, 25°58′26″ N, 98°42′23″ E, altitude 2590–2690 m, 6 August 2022, GLG-FXP456 (KUN-HKAS144472).

Notes: *Gymnopus yunnanensis* is characterized by its often yellowish brown pileus disc and hygrophanous margin, a dark-colored stipe without rhizomorphs at the base, oblong basidiospores measuring 5–6.5 × 2.5–3 μm, and large cheilocystidia. In the molecular phylogenetic analysis (Figure 1), *G*. *yunnanensis* is closely related to the clade formed by *G*. *aurantiofuscus* and *G. viridocephalus*. However, *G. aurantiofuscus*, originally described from southwestern China, has a darker-colored basidioma with stubbier cheilocystidia (13.3–31.1 × 3.6–10.6 μm), along with the presence of pleurocystidia [29]. *Gymnopus viridocephalus* was also described from the studied area but differs from *G. yunnanensis* by its smaller basidioma with yellowish green pileus (pileus 20.5–26.5 mm, stipe 51–68 × 1.8–2.9 mm), and the presence of pleurocystidia [29]. Morphologically, *G*. *yunnanensis* looks like *G*. *dryophiloides*, a species distributed in the Republic of Korea and China. However, *G*. *dryophiloides* differs from *G. yunnanensis* by its basidioma with whitish to pinkish ochraceous rhizomorphs at the stipe base, and ellipsoid to ellipsoid-fusoid, or pip-shaped basidiospores [23].

## 4. Discussion

Species of *G*. sect. *Levipedes* are distributed worldwide. To date, a total of 30 species have been recorded in China, including the previously reported 26 species, and three new species described in this study: *G*. *sinobrevipes*, *G. flavoalbus* and *G*. *yunnanensis*. Additionally, one species, *G. bicolor*, which is new to China, was also discovered during this research. However, it is important to note that many species records in China, especially those originally described from Europe and North America, still need further verification. For example, the sample (HMAS269405), collected from Qinghai, China, was identified as *G. dryophilus* (Bull.) Murrill. However, it was proved to be *G*. *dryophiloides* in our analyses (Figure 1). The other collections from southwestern China, which were previously identified as *G. dryophilus*, have also been confirmed to be *G*. *dryophiloides*. In addition, the distributions of *G*. *erythropus* and *G. aquosus* (Bull.) Antonín and Noordel in China remain uncertain because the samples from China and European countries did not form a monophyletic group (Figure 1).

A total of 17 *Gymnopus* species have been recorded in southwestern China [26,28,29,31,34,36,37,40,41,42,43,45]. This includes three species new to science, two species new to the studied area (*G. bicolor* and *G. ocior*), and 12 known species. However, among the 17 known species, the distributions of six species in the area lack molecular evidence and require further verification. Four of these six species were originally described from Europe: *G. aquosus*, *G. erythropus*, *G. dryophilus*, and *G. fuscopurpureus* (Pers.) Antonín, Halling and Noordel [3,12,56]. The remaining two species, *G. alkalivirens* (Singer) Halling and *G. brunneodiscus* Antonín, Ryoo, and Ka, were originally described from North America and the Republic of Korea, respectively [14,23]. According to our phylogenetic analyses, only *G*. *ocior*, originally described from Europe, was found in southwestern China. Compared to the other parts of China, the species diversity of *G*. sect. *Levipedes* in the southwestern region is relatively high, which may be due to the region’s complex topography and geography, variable climate, and luxuriant vegetation.

The color of the pileus and lamellae, the shape and size of basidiospores, and the shape of cheilocystidia are important characteristics for species delimitation in *G.* sect. *Levipedes* [12,56]. In southwestern China, most species of the section have white lamellae. However, the lamellae of *G*. *dryophiloides* and *G*. *ocior* vary from white to yellow [26,27,28,29,30]. The shape of cheilocystidia is a good characteristic for species recognition [56]. Among the 11 species confirmed from southwestern China, *G. sinobrevipes* and *G*. *strigosipes* [26] possess clavate to captitate cheilocystidia, whereas other species possess furcate and lobate cheilocystidia. The presence of pleurocystidia is also a good characteristic for species delimitation. Li et al. [29] reported that *G*. *aurantiofuscus* and *G*. *viridocephalus* possess fusiform pleurocystidia; however, pleurocystidia were not observed in the other species reported from southwestern China [12,17,23,26,28]. In addition, the presence of incrusted hyphae in the pileipellis can also be used to distinguish species of the section. For example, *G*. *aurantiofuscus*, *G*. *bicolor*, and *G*. *ocior* can be distinguished from other species reported from the studied area by the presence of encrusted hyphae in their pileipellis [12,17,23,26,28,29].

For comparison and identification, a key to the 11 species of *G*. sect. *Levipedes* with morpho-molecular evidence from southwestern China is provided below, highlighting these features in identifying the species.

**Key to the Species of *Gymnopus* sect. *Levipedes* with morpho-molecular evidence from southwestern China**

1. Stipe base with rhizomorphs, or with wiry mycelium………………………………………………………………………………………………………………………………………………………………………………………….….2
1′. Stipe base without rhizomorphs or wiry mycelium, or sparsely covered with tomentum…………………….………………………………………………………………………………………………………………………….….6
2. Lamellae white to yellow, edges serrulate or fimbriate……………………………………………………………………………………………………………………………………………………………………………………………3
2′. Lamellae white, edges entire and without fimbriate………………………………………………………………………………………………………………………………………………………………………………………………4
3. Pileus pale yellow to orange or watery ochraceous yellow; pileipellis hyphae smooth or incrusted, pale yellow-brown in alkali………………………………………………………………………………………………………*G*. *dryophiloides*
3′. Pileus dark, reddish brown to chestnut, fading upon drying to buff; pileipellis hyphae with lightly olivaceous brown incrustations in alkali, occasionally with scattered amorphous brownish pigment globules……*G*. *ocior*
4. Stipe short (30–42 μm long); pileipellis hyphae with annular incrustations……………………………………………………………………………………………………………………………………………………………………*G*. *bicolor*
4′. Stipe longer (30–90 μm long); pileipellis hyphae smooth…………………………………………………………………………………………………………………………………………………………………………………………5
5. Stipe with lighter color, yellowish white to yellowish brown; basidiospores 5–6.5 × 2.5–3 μm; cheilocystidia 15–57 × 2–6 μm…………………………………………………………………………………………………………*G*. *yunnanensis*
5′. Stipe with darker color, pinkish brown to rusty brown or brown; basidiospores 6–8 × 3–4 μm; cheilocystidia 16–33 × 3–8 μm…………………………………………………………………………………………………….….*G*. *flavoalbus*
6. Cheilocystidia clavate to captitate, not furcate………………………………………………………………………………………………………………………………………………………………………………………………………7
6′. Cheilocystidia coralloid, often irregular, lobed……………………………………………………………………………………………………………………………………………………………………………………………….….…8
7. Stipe 40–85 × 3–6 mm; gregarious on the leaf litter or rotten branches in coniferous forest dominated by *Pinus*……………………………………………………………………………………………………………………………*G*. *jilongensis*
7′. Stipe 15–40 × 1–3 mm; scattered at the base of trees or on the leaf litter in subtropical broad-leaved forest…………………………………………………………………………………………………………………………………*G*. *sinobrevipes*
8. Stipitipellis hyphae with brown granules on cell walls; stipitipellis hyphae and caulocystidia staining green in KOH……………………………………………………………………………………………………………………*G*. *stipitovirens*
8′. Stipitipellis hyphae lacking brown granules on cell walls; stipitipellis hyphae and caulocystidia/terminal cells not staining green in KOH…………………………………………………………………………………………8
9. Pleurocystidia present, fusiform………………………………………………………………………………………………………………………………………………………………………………………………………………………10
9′. Pleurocystidia absent……………………………………………………………………………………………………………………………………………………………………………………………………………………………………*G*. *strigosipes*
10. Pileus orange-grey to greyish orange overall when young, finally dark brown or gradually fading towards margin to almost orange-white; stipitipellis composed of cylindrical hyphae, with scattered diverticula…*G*. *aurantiofuscus*
10′. Pileus yellowish green when very young, finally olive brown; stipitipellis composed of cylindrical hyphae, without diverticula………………………………………………………………………………………………….…*G*. *viridocephalus*

## Figures and Tables

**Table 1:** Information of specimens used in phylogenetic analyses and their GenBank accession numbers. Sequences newly generated in this study are indicated in bold.

Species	Voucher	Locality	GenBank Accession Numbers		Reference
			ITS	LSU	
*Gymnopus alkalivirens*	TENN 51249	USA	DQ450000	–	[18]
*G. alpinus*	Duke14	UK	DQ480102	–	[18]
*G. alpinus*	Duke15	Sweden	DQ480101	–	[18]
*G. aquosus*	BRNM 710027	Czech Republic	JX536170	–	[56]
*G. aquosus*	BRNM 670755	Czech Republic	JX536171	–	[56]
*‘* *G.* *a* *quosus* *’*	HMAS 293906	China	PP741435	–	[42]
*G. aurantiipes*	AWW118	Indonesia	AY263432	AY639410	[17,57]
*G. aurantiipes*	AWW131	Indonesia	AY263433	–	[17]
*G. aurantiofuscus*	HGASMF01–7019	China	PP151498	PP151550	[29]
*G. aurantiofuscus*	HGASMF01–7024	China	PP151504	PP151551	[29]
*G. austrosemihirtipes*	AWW65	Indonesia	AY263422	–	[17]
*G. bicolor*	AWW116	Indonesia	AY263423	AY639411	[17]
***G. bicolor***	**KUN-HKAS144469**	**China**	**PQ771157**	**PQ771142**	**This study**
*G. bisporus*	SCM B-4065	Spain	JN247551	JN247555	[22]
*G. brunneodiscus*	BRNM 808975	Republic of Korea	MH589975	MH589991	[23]
*G. brunneodiscus*	BJTC FM675	China	OK662958	–	[30]
*G. brunneodiscus*	BRNM 714974	Republic of Korea	MH589973	MH589988	[42]
*G. catalonicus*	SCM B-4057	Spain	JN247552	JN247556	[22]
*G*. *changbaiensis*	HMJAU60300	China	OM030272	OM033387	[25]
*G*. *changbaiensis*	HMJAU60302	China	OM030274	OM033389	[25]
***G. dryophiloides***	**KUN-HKAS84656**	**China**	**PQ771160**	**PQ771145**	**This study**
***G. dryophiloides***	**KUN-HKAS124802**	**China**	**PQ771158**	**PQ771143**	**This study**
***G. dryophiloides***	**KUN-HKAS83544**	**China**	**PQ771159**	**PQ771144**	**This study**
*G. dryophiloides*	MJ 160	China	OR711256	OR711258	[45]
*G. dryophiloides*	BRNM 781447	Republic of Korea	MH589967	MH589985	[23]
*G. dryophiloides*	BJTC FM520	China	OK662665	–	[30]
*G. dryophilus*	DUKE 193411	Sweden	JX536153	–	[56]
*G. dryophilus*	BRNM 707149	Italy	JX536157	–	[56]
*‘* *G. dryophilus* *’*	HMAS269405	China	PP741403	–	[42]
*G. earleae*	TENN 59457	USA	AY256694	–	[58]
*‘* *G. erythropus* *’*	HMJAU60315	China	OM030280	OM033396	[25]
*‘* *G. erythropus* *’*	BJTC FM1602	China	OK663002	–	[30]
*G. erythropus*	BRNM 705224	Czech Republic	JX536131	–	[56]
*G. erythropus*	BRNM 693553	Switzerland	JX536135	–	[56]
*G. exculptus*	TFB 10718	Greenland	DQ449973	–	[18]
*G. fagiphilus*	BRNM 707068	Czech Republic	JX536130	–	[56]
*G. fagiphilus*	BRNM 695747	Slovakia	JX536127	–	[56]
***G*** **. *flavoalbus***	**KUN-HKAS144470**	**China**	PQ771152	PQ771137	**This study**
***G*** **. *flavoalbus***	**KUN-HKAS144471**	**China**	PQ771153	PQ771138	**This study**
*G. fuscopurpureus*	BRNM 809119	Czech Republic	MZ542559	MZ542563	[24]
*G. fusipes*	TENN 59300	Austria	AY256711	AY256711	[58]
*G. globulosus*	HMJAU60307	China	OM030269	OM033406	[25]
*G. globulosus*	HMJAU60308	China	OM030270	OM033407	[25]
*G. hybridus*	BRNM 695773	Italy	JX536177	–	[56]
*G. hybridus*	TENN 59315	Austria	DQ449980	–	[18]
*G. indoctoides*	AWW125	Indonesia	AY263424	AY639419	[17]
*G. inexpectatus*	MCVE 16126	Italy	EU622905	EU622906	[21]
*G. inusitatus*	SCM B-4058	Spain	JN247553	JN247557	[22]
*G. inusitatus* var. *cystidiatus*	BRNM 737257	Hungary	JN247550	JN247554	[22]
*G. jilongensis*	HMAS 300549	China	PQ099856	PP968805	[28]
*G. junquilleus*	TENN 55224	USA	DQ449969	–	[18]
*G. kauffmanii*	Duke230	USA	DQ450001	–	[18]
*G. lanipes*	BRNM 670686	Spain	JX536137	–	[56]
*G. loiseleurietorum*	URM 90060	Sweden	KY321571	KY321572	Direct sub.
*G. longisterigmaticus*	HMJAU60288	China	OM030282	OM033403	[25]
*G. longistipes*	HMJAU61076	China	PP646156	PP646168	[27]
*G. longistipes*	HMJAU61200	China	PP646157	PP646169	[27]
*G. longus*	HMJAU60291	China	OM030285	OM033400	[25]
*G. longus*	HMJAU60293	China	OM030287	OM033402	[25]
*G. macropus*	TENN 56636	Costa Rica	DQ449978	–	[18]
*G. macropus*	TENN 58619	Costa Rica	DQ449979	–	[18]
*G. macrosporus*	HMJAU60294	China	OM030266	OM033397	[25]
*G. macrosporus*	HMJAU60295	China	OM030267	OM033398	[25]
*G. nigrescens*	TFC Mic. 25409	Spain	MZ542560	MZ542560	[24]
***G. ocior***	**KUN-HKAS83528**	**China**	**PQ771161**	**PQ771146**	**This study**
*G. ocior*	BRNM 728540	Czech Republic	JX536161	–	[56]
*G. ocior*	BRNM 737697	Norway	JX536162	–	[56]
*G. ocior*	HMAS296502	China	PP741445	–	[42]
*G. omphalinoides*	GDGM 78318	China	MW134044	MW134730	[59]
*G. pubipes*	AH 26931	Spain	MZ542558	MZ542562	[24]
*G. schizophyllus*	GDGM 77165	China	MW134043	MW134729	[59]
*G. semihirtipes*	TENN: F 07595	USA	OK376741	–	Direct sub.
*G. sepiiconicus*	AWW126	Indonesia	AY263449	AY639427	[17,57]
*G. sepiiconicus*	AWW117	Indonesia	AY263448	–	[17]
*G. similis*	BRNM 766739	Republic of Korea	KP336692	KP336699	[60]
***G*** **.** ***sino*** ***brevipe*** ***s***	**KUN-HKAS107802**	**China**	PQ771151	PQ771136	**This study**
***G*** **.** ***sino*** ***brevipe*** ***s***	**KUN-HKAS108042**	**China**	PQ771149	PQ771134	**This study**
***G*** **.** ***sino*** ***brevipe*** ***s***	**KUN-HKAS108101**	**China**	PQ771150	PQ771135	**This study**
*G. spongiosus*	TENN 59640	USA	DQ480113	–	[18]
*G. spongiosus*	TENN: F-68184	USA	KY026706	KY026706	[9]
*G. stipitovirens*	HMJAU60420	China	PP151528	–	[29]
*G. stipitovirens*	HMJAU60423	China	PP151524	–	[29]
*G. stipitovirens*	BRNM 718669	Republic of Korea	MH589976	MH589993	[23]
*G. striatipileatus*	HMJAU61073	China	PP646164	PP646176	[27]
*G. striatipileatus*	HMJAU61074	China	PP646165	PP646177	[27]
*G. striatus*	HMJAU60297	China	OM030263	OM033384	[25]
*G. strigosipes*	HMAS 295796	China	OM970874	OM970874	[26]
*G. strigosipes*	HMAS 295797	China	OM970867	OM970867	[26]
*G. subsulphureus*	TENN 56321	USA	DQ449972	–	[18]
*G. tiliicola*	HMJAU60304	China	OM030275	OM033392	[25]
*G. tiliicola*	HMJAU60305	China	OM030276	OM033394	[25]
*G. tomentosus*	HMJAU60303 (1)	China	OM030278	OM033390	[25]
*G. tomentosus*	HMJAU60303 (2)	China	OM030279	OM033391	[25]
*G. variicolor*	BRNM 781307	Republic of Korea	KX926134	–	[60]
*G. viridiscus*	HMJAU61202	China	PP646159	PP646171	[27]
*G. viridiscus*	HMJAU61203	China	PP646163	PP646175	[27]
*G. viridocephalus*	HGASMF01–7003	China	PP151513	PP151559	[29]
***G. viridocephalus***	**KUN-HKAS132571**	**China**	**PQ771162**	**PQ771147**	**This study**
***G. viridocephalus***	**KUN-HKAS132589**	**China**	**PQ771163**	**PQ771148**	**This study**
*G. vitellinipes*	AWW115	Indonesia	AY263453	–	[17]
*G. vitellinipes*	AWW127	Indonesia	AY263429	AY639432	[17,57]
***G. yunnanensis***	**KUN-HKAS108570**	**China**	**PQ771154**	**PQ771139**	**This study**
***G. yunnanensis***	**KUN-HKAS108610**	**China**	**PQ771155**	**PQ771140**	**This study**
***G. yunnanensis***	**KUN-HKAS144472**	**China**	**PQ771156**	**PQ771141**	**This study**
*Gymnopus* sp.	PUL00037691	USA	ON561598	–	Direct sub.
*Gymnopus* sp.	HAY-F-003846	USA	PQ144058	–	Direct sub.
*Gymnopus* sp.	HAY-F-006720	USA	PP335760	–	Direct sub.

Quotation marks are added to indicate the uncertain taxonomic positions; – represents missing corresponding sequences.

## Data Availability

Publicly available datasets were analyzed in this study (https://www.ncbi.nlm.nih.gov/, accessed on 17 December 2024; https://nmdc.cn/fungalnames/, accessed on 15 December 2024).

## References

[1] He M.Q., Zhao R.L., Hyde K.D., Begerow D., Kemler M., Yurkov A., McKenzie E.H.C., Raspé O., Kakishima M., Sánchez-Ramírez S. (2019). Notes, outline and divergence times of Basidiomycota. Fungal Divers..

[2] Kalichman J., Kirk P.M., Matheny P.B. (2020). A compendium of generic names of agarics and Agaricales. Taxon.

[3] Persoon C. (1801). Synopsis Methodica Fungorum [A Methodical Synopsis of the Fungi].

[4] Roussel H.-F.-A. (1806). Flore du Calvados et Terrains Adjacents, Composée Suivant la Méthode de Jussieu.

[5] Antonín V., Halling R.E., Noordeloos M.E. (1997). Generic concepts within the groups of Marasmius and Collybia sensu lato. Mycotaxon.

[6] Antonín V., Noordeloos M.E. (1997). A Monograph of Marasmius, Collybia and Related Genera in Europe. Part 2: Collybia, Gymnopus, Rhodocollybia, Crinipellus, Chaetocalathus and Additions to Marasmiellus.

[7] Noordeloos M.E., Antonín V. (2008). Contribution to a monograph of marasmioid and collybioid fungi in Europe. Czech Mycol..

[8] Antonín V., Noordeloos M.E. (2010). A Monograph of Marasmioid and Collybioid Fungi in Europe.

[9] Petersen R.H., Hughes K.W. (2016). *Micromphale* sect. *Perforantia* (Agaricales, Basidiomycetes); Expansion and phylogenetic placement. MycoKeys.

[10] Oliveira J.J.S., Vargas-Isla R., Cabral T.S., Rodrigues D.P., Ishikawa N.K. (2019). Progress on the phylogeny of the Omphalotaceae: *Gymnopus* s. str., *Marasmiellus* s. str., *Paragymnopus* gen. nov. and *Pusillomyces* gen. nov. Mycol. Prog..

[11] Vilgalys R., Miller O.K. (1983). Biological Species in the *Collybia Dryophila* Group in North America. Mycologia.

[12] Vilgalys R., Miller O.K. (1987). Morphological studies on the *Collybia dryophila* group in Europe. Trans. Br. Mycol. Soc..

[13] Vilgalys R. (1991). Speciation and Species Concepts in the *Collybia dryophila* Complex. Mycologia.

[14] Halling R.E. (1981). Notes on *Collybia*. II. Additional Taxa That Are Green in Alkaline Solution. Mycologia.

[15] Halling R.E. (1996). Notes on *Collybia* V. *Gymnopus* section *Levipedes* in tropical South America, with comments on Collybia. Brittonia.

[16] Ortega A., Antonín V., Esteve-Raventós F. (2003). Three interesting thermophilic taxa of *Gymnopus* (Basidiomycetes, Tricholomataceae): *G. pubipes* sp. nov., *G. pubipes* var. *pallidopileatus* var. nov. and *G. dryophilus* var. *lanipes* comb. nov. Mycotaxon.

[17] Wilson A.W., Desjardin D.E., Horak E. (2004). Agaricales of Indonesia. 5. The genus *Gymnopus* from Java and Bali. Sydowia.

[18] Mata J.L., Hughes K.W., Petersen R.H. (2006). An investigation of /omphalotaceae (Fungi: Euagarics) with emphasis on the genus *Gymnopus*. Sydowia.

[19] Vila J., Llimona X. (2006). Noves dades sobre el component fúngic de les comunitats de Cistus de Catalunya. Il. Rev. Catalana Micol..

[20] Polemis E., Noordeloos M.E. (2007). Two new *Gymnopus* species from the Island of Andros (Kiklades, C. Aegean, Greece). Mycotaxon.

[21] Vizzini A., Consiglio G., Antonín V., Marco C. (2008). A new species within the *Gymnopus dryophilus* complex (Agaricomycetes, Basidiomycota) from Italy. Mycotaxon.

[22] Antonín V., Finy P., Tomšovský M. (2012). Taxonomy of the *Gymnopus inusitatus* group and the new *G. inusitatus* var. *cystidiatus* from Hungary. Mycotaxon.

[23] Ryoo R., Antonin V., Ka K.-H. (2020). Marasmioid and Gymnopoid Fungi of the Republic of Korea. 8. *Gymnopus* Section *Levipedes*. Mycobiology.

[24] Crous P.W., Osieck E.R., Jurjevic Z., Boers J., van Iperen A.L., Starink-Willemse M., Dima B., Balashov S., Bulgakov T.S., Johnston P.R. (2021). Fungal Planet description sheets: 1284–1382. Persoonia.

[25] Hu J.J., Zhao G.P., Tuo Y.L., Rao G., Zhang Z.H., Qi Z.X., Yue L., Liu Y.J., Zhang T., Li Y. (2022). Morphological and Molecular Evidence Reveal Eight New Species of *Gymnopus* from Northeast China. J. Fungi.

[26] Li J.P., Pan M.C., Li Y., Deng C.Y., Wang X.M., Zhang B.X., Li C.T., Li Y. (2022). Morpho-Molecular Evidence Reveals Four Novel Species of *Gymnopus* (Agaricales, Omphalotaceae) from China. J. Fungi.

[27] Hu J.J., Tuo Y.L., Qi Z.X., Li X.F., Jiang D.H., Zhang B., Li Y. (2024). The Combination of Morphological and Phylogenetic Evidence Reveals Four New *Gymnopus* Species and New Distribution. J. Fungi.

[28] Wang K., Liu S.L., Liu X.Z., Hong P., Wei H.W., Wang Y., Phurbu D., Zhou L.W., Wei T.Z. (2024). Catalogue of fungi in China 3. New taxa of macrofungi from southern Xizang, China. Mycology.

[29] Li J.P., Oliveira J.J.S., Pan M.C., Deng C.Y., Antonín V., Xiao Z.D., Li F.F., Li T.H., Li C.T., Dai Y.T. (2024). Notes on all Genera of Omphalotaceae: Expanding the Taxonomic Spectrum in China and Revisiting Historical Type Specimens. Mycosphere.

[30] Mao N., Liu H., Fan L.I. (2022). *Gymnopus wutaishanensis* (Omphalotaceae, Agaricales) a new species from North China. Phytotaxa.

[31] Mao X.L. (1998). Economic Fungi of China.

[32] Qi L.L. (2016). Studies on Macrofungi Diversity in Larch Forest of Northeastern China. Ph.D. Thesis.

[33] Li Y., Li T.H., Yang Z.L., Dai Y.C., Bau T. (2015). Atlas of Chinese Macrofungal Resources.

[34] Ying J.Z., Zang M. (1994). The Macrofungi in Southwestern China.

[35] Wang W., Bau T. (2015). Diversity of Mycobiota and Ecological Distribution of Macrofungi in Changbai Mountain. J. Jilin Agric. Univ..

[36] Environment Protection Administration of Yunnan Province, Kunming Branch Chinese Academy of Sciences (2016). Checklist of Biological Species of Yunnan Province.

[37] Mao X.L. (2000). The Macrofungi in China.

[38] Zhang P. (2017). Diversity of Macrofungi in the Greater and Lesser Khinggan Moutains. Ph.D. Thesis.

[39] Tuo Y.L., Na R., Hu J.J., Zhao G.P., Wang Y., Zhang Z.H., Qi Z.X., Li Y., Zhang B. (2022). Exploring the Relationships between Macrofungi Diversity and Major Environmental Factors in Wunvfeng National Forest Park in Northeast China. J. Fungi.

[40] Mu M. (2021). Diversity of Macrofungi in Junzi Shan. Master’s Thesis.

[41] Chen A.M. (2022). Survey of Macrofungi Diversity and *Lactaricus* Genetic Diversity in Suiyang County. Master’s Thesis.

[42] Wang K., Du Z., Hong P., Zhao M.J., Li G.j., Yu X.D., Wei T.Z. (2024). Taxonomy of *Collybiopsis* spp. and *Gymnopus* spp. in China. J. Liaocheng Univ. (Nat. Sci. Ed.).

[43] Yang Z.L., Wang X.H., Wu G. (2022). Mushrooms of Yunnan.

[44] Bau T., Kang G.P., Fan Y.G., Wang Y., Liang H. (2011). Checklist of macrofungi collected from different forests in Changbai Mountain (IV): Coniferous and broad-leaved mixed forest. Journal of Fungal research. J. Fungal Res..

[45] Ma J., Liu H.M., Yu T.J., Yang M., Tang L.P. (2024). A Poisoning Case Involving *Gymnopus dryophiloides* (Agaricomycetes). Int. J. Med. Mushrooms.

[46] Gardes M., Bruns T.D. (1993). ITS primers with enhanced specificity for basidiomycetes—Application to the identification of mycorrhizae and rusts. Mol. Ecol..

[47] Vilgalys R., Hester M. (1990). Rapid Genetic Identification and Mapping of Enzymatically Amplified Ribosomal DNA from Several *Cryptococcus* Species. J. Bacteriol..

[48] Katoh K., Standley D.M. (2013). MAFFT multiple sequence alignment software version 7: Improvements in performance and usability. Mol. Biol. Evol..

[49] Katoh K., Rozewicki J., Yamada K.D. (2019). MAFFT online service: Multiple sequence alignment, interactive sequence choice and visualization. Brief. Bioinform..

[50] Hall T.A. (1999). BioEdit: A User-Friendly Biological Sequence Alignment Editor and Analysis Program for Windows 95/98/NT. Nucleic Acids Symp. Ser..

[51] Zhang D., Gao F., Jakovlić I., Zou H., Zhang J., Li W.X., Wang G.T. (2020). PhyloSuite: An integrated and scalable desktop platform for streamlined molecular sequence data management and evolutionary phylogenetics studies. Mol. Ecol. Resour..

[52] Xiang C.Y., Gao F., Jakovlic I., Lei H.P., Hu Y., Zhang H., Zou H., Wang G.T., Zhang D. (2023). Using PhyloSuite for molecular phylogeny and tree-based analyses. Imeta.

[53] Posada D., Crandall K.A. (1998). MODELTEST: Testing the model of DNA substitution. Bioinformatics.

[54] Edler D., Klein J., Antonelli A., Silvestro D., Matschiner M. (2020). raxmlGUI 2.0: A graphical interface and toolkit for phylogenetic analyses using RAxML. Methods Ecol. Evol..

[55] Ronquist F., Teslenko M., van der Mark P., Ayres D.L., Darling A., Hohna S., Larget B., Liu L., Suchard M.A., Huelsenbeck J.P. (2012). MrBayes 3.2: Efficient Bayesian phylogenetic inference and model choice across a large model space. Syst. Biol..

[56] Antonín V., Sedlák P., Tomšovský M. (2013). Taxonomy and phylogeny of European *Gymnopus* subsection *Levipedes* (Basidiomycota, Omphalotaceae). Persoonia.

[57] Wilson A.W., Desjardin D.E. (2005). Phylogenetic relationships in the gymnopoid and marasmioid fungi (Basidiomycetes, euagarics clade). Mycologia.

[58] Mata J.L., Hughes K.W., Petersen R.H. (2004). Phylogenetic placement of *Marasmiellus juniperinus*. Mycoscience.

[59] Li J.P., Antonín V., Gates G., Jiang L., Li T.H., Li Y., Song B., Deng C.Y. (2022). Emending *Gymnopus* sect. *Gymnopus* (Agaricales, Omphalotaceae) by including two new species from southern China. MycoKeys.

[60] Ryoo R., Antonín V., Ka K.H., Tomšovský M. (2016). Marasmioid and gymnopoid fungi of the Republic of Korea. 8. *Gymnopus* section Impudicae. Phytotaxa.

